# Novel innovations in cell and gene therapies for spinal cord injury

**DOI:** 10.12688/f1000research.21989.1

**Published:** 2020-04-22

**Authors:** Mohammad-Masoud Zavvarian, Amirali Toossi, Mohamad Khazaei, James Hong, Michael Fehlings

**Affiliations:** 1 Krembil Research Institute, University Health Network, Toronto, Canada; 2 Institute of Medical Science, University of Toronto, Toronto, Canada; 3 Department of Surgery, University of Toronto, Toronto, Canada; 4 Spinal Program, Toronto Western Hospital, University Health Network, Toronto, Canada

**Keywords:** Spinal Cord Injury; Gene Therapy; Neuroregeneration; Stem Cells; Combinatorial Treatments

## Abstract

Spinal cord injury (SCI) leads to chronic and multifaceted disability, which severely impacts the physical and mental health as well as the socio-economic status of affected individuals. Permanent disabilities following SCI result from the failure of injured neurons to regenerate and rebuild functional connections with their original targets. Inhibitory factors present in the SCI microenvironment and the poor intrinsic regenerative capacity of adult spinal cord neurons are obstacles for regeneration and functional recovery. Considerable progress has been made in recent years in developing cell and molecular approaches to enable the regeneration of damaged spinal cord tissue. In this review, we highlight several potent cell-based approaches and genetic manipulation strategies (gene therapy) that are being investigated to reconstruct damaged or lost spinal neural circuits and explore emerging novel combinatorial approaches for enhancing recovery from SCI.

## Introduction

Traumatic spinal cord injury (SCI) can prompt debilitating autonomic and sensorimotor impairments, severely limiting the patient’s independence, quality of life, and socioeconomic status ^1, 2^. The prevalence of SCI ranges between 250 and 906 cases per million, with life expectancy typically spanning several decades from the time of injury ^3^. This leads to a prolonged life with disabilities and a staggering cost of care (estimated between 1.2 and 5.1 million US dollars for the patient’s lifespan) ^4^. In addition, concurrent complications, such as respiratory difficulty ^5^, autoimmune dysfunction ^6^, neuropathic pain ^7^, and autonomic dysreflexia ^8^, further exacerbate the patient’s health and wellbeing. While many regenerative treatments have been investigated over the years, an effective therapy does not yet exist in the clinic ^2^. This paper reviews several emerging regenerative strategies for SCI and the knowledge gaps associated with them. These treatment strategies are 1) regenerative genetic manipulation (gene therapy) approaches, 2) cellular transplantation therapy, and 3) combinatorial approaches to enhance functional outcomes.

## Pathological hallmarks of spinal cord injury

The traumatic fracture and dislocation of the vertebral column introduces laceration, compression, and contusive damage to the spinal cord, impairing local neurons and the supporting glial and vascular cells ^9^. The primary mechanical shock to the neural tissue and disruption of the cell membrane during the primary injury permeabilizes the cells, resulting in a cascade of molecular and signaling pathways that initiate a series of secondary injuries to the spinal cord ^10^. The formation of free radicals and oxidative stress as a consequence of secondary injuries result in more neuronal and glial death, mainly due to apoptosis. This also results in activation of local microglia and astrocytes to produce proinflammatory signals for a greater immune response ^11^. In parallel, the acute vascular damage and permeabilization will increase hemorrhage, hypoxia, and the infiltration of reactive immune cells to the injury epicenter. Despite the adaptive role of the introduced immune cells in debris clearance, the prolonged activity of immune cells leads to swelling and further damages the local cells ^10^.

The injured spinal cord tissue can be divided into three distinct histological compartments, including a non-neural lesion core, a surrounding astroglial border, and a preserved reactive neural tissue ^12^. Each compartment is composed of a unique cellular makeup and poses a distinct barrier to functional recovery ^13^. First, the lesion core is the site of the fibrotic scar and cystic cavity ^14^. Cavitation results from debris clearance but limits axonal growth and neurogenesis ^12^. The surrounding astroglial scar is formed from newly differentiated astrocytes, which encapsulate the reactive immune cells within the damaged tissue zone. The functional role of astrocytic production and the integration of inhibitory molecules within the astroglial border continues to be under investigation. While genetic knockout studies demonstrate the adaptive role of astrocyte scar formation ^15^, the prevailing paradigm suggests that this border constitutes a major impediment to neural regeneration ^16^. In the perilesional zone, synaptic damage and demyelination result in circuit inactivation ^17^. Circuit reorganization can be either maladaptive—leading to neuropathic pain, muscle spasticity, and autonomic dysreflexia—or adaptive, as it can restore function after incomplete SCI ^12^.

**Figure 1.**
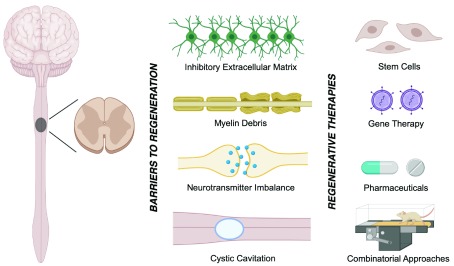
Spinal cord injury pathology and regenerative therapeutics. The endogenous attempts for recovery following traumatic spinal cord injury (SCI) are hindered by 1) inactivated regenerative pathways within neurons and their progenitor cells, 2) myelin debris and the associated inhibitory molecules, such as neurite outgrowth inhibitor (Nogo), myelin-associated glycoprotein (MAG), and oligodendrocyte-myelin glycoprotein (OMgp), 3) the formation of a cystic cavity, and 4) the inhibitory extracellular matrix. Cell and gene therapies are emerging treatment strategies for traumatic SCI.

During both primary and secondary injury, the secretion of inhibitory molecules is a critical barrier to regeneration. For instance, disintegration of myelin and demyelination releases potent inhibitory extracellular molecules, such as myelin-associated glycoprotein (MAG), oligodendrocyte-myelin glycoprotein (OMgp), and neurite outgrowth inhibitor A (Nogo A) ^18, 19^. Furthermore, reactive glia secrete tenascin as well as chondroitinase sulfate proteoglycans (CSPGs), which include brevican, phosphacan, neurocan, versican, and neural/glial antigen 2 (NG2) proteoglycans ^20^. These molecules lead to the activation of the Rho–ROCK signaling pathway, which intrinsically inhibits neuronal repair and regeneration ^21^. Hence, despite the wide distribution of oligodendrocyte progenitor cells (OPCs) and the localization of neural progenitor cells (NPCs) along the ependymal layers of the spinal central canal, limited endogenous neural regeneration occurs in the injured spinal cord ^22, 23^. Numerous therapeutics aimed at mitigating these barriers to regeneration have been examined over the past few decades in both clinical and preclinical studies ^24– 27^ ( Figure 1). This review provides a concise overview of the newly developed regenerative gene and cell therapies following traumatic SCI.

## Regenerative gene therapy

**Figure 2.**
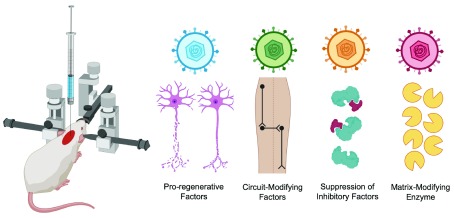
Gene therapy applications investigated in preclinical spinal cord injury models. Adeno-associated viruses (AAVs) introduce non-integrating genetic material, which can express 1) pro-regenerative factors, 2) circuit-modifying factors, 3) gene silencers for inhibitory factors, and 4) matrix-modifying enzymes..

Gene therapy is the introduction of new genetic material to modify maladaptive transcription in a cell or to introduce downregulated or novel genes ^28^. The advent of CRISPR/Cas9 genome editing approaches and recombinant replication-defective viral constructs enables targeted interventions in the injured spinal cord, mitigating the risk of potential adverse off-target effects. Gene therapy has gained promising advancement in the past decade, as six therapies have gained clinical approval for conditions such as spinal muscular atrophy or Leber’s congenital amaurosis ^29^. Two potential applications of gene therapy for SCI include *in vivo* gene delivery to the spinal cord or *ex vivo* transduction of cells for subsequent transplantation into the spinal cord. The advancement of *in vivo* gene delivery via non-integrating adeno-associated viral (AAV) constructs allows durable and sustained episomal expression of a therapeutic gene or a gene silencer ( Figure 2) ^30^. Thus far, preclinical investigations reveal the applications of *in vivo* gene therapy in the injured spinal cord to 1) enhance the expression of pro-regenerative factors, 2) molecularly modulate neural circuits, 3) block the expression of detrimental proteins, and 4) introduce matrix-modifying enzymes for the degradation of inhibitory particles.

### Expression of pro-regenerative factors

The injured axons at the lesion core possess limited regenerative ability. The expression of pro-regenerative factors can increase the regenerative potential of damaged neurons. Krüppel-like factors (KLFs) are a family of transcriptional factors which are crucial for axonal regeneration and plasticity. Although KLF4 inhibits axon regeneration, KLF6 and KLF7 are important promoters of axon regeneration ^31, 32^. Therapeutically induced KLF7 overexpression stimulates axonal sprouting ^32^. Another pro-regenerative gene therapy target is SOX11, which is a transcriptional factor actively involved in neurogenesis. SOX11 overexpression via an AAV-mediated strategy promotes axonal sprouting in preclinical SCI models ^33^. There is growing evidence that combined overexpression of growth factors will have a greater effect on axonal regeneration. Multiple genes can be combined into the same viral construct, which ease their therapeutic administration. The combined AAV-induced overexpression of osteopontin, insulin-like growth factor 1 (IGF1), ciliary-derived neurotrophic factor (CNTF), fibroblast growth factor 2 (FGF2), glial-derived neurotrophic factor (GDNF), and epidermal growth factor (EGF) suggests a 100-fold increase in axonal growth ^34^.

### Expression of circuit-modifying factors

The majority of SCI patients suffering from complete functional loss (classified as grade A by the American Spinal Injury Association) continue to possess anatomically preserved neural tissue around the lesion core, which remains dormant after injury ^17, 35^. Neural circuit modulation utilizes the neuroplastic nature of local synapses to reform functional “bypass” circuits around the lesion core, hence activating the dormant preserved neural tissue ^36^. The staggered double hemisection (SDH) SCI model enables the examination of local relay circuits, as it interrupts all supraspinal inputs while sparing contralateral relay connections in the spinal cord ^37^. Although advances in rehabilitative training and epidural stimulation have shown incremental progress in stimulating dormant circuits, these therapies can be strengthened and supplemented with molecular modulators of relay circuits to maximize their effects. Recent pharmacological screening in mouse SDH has identified chloride potassium symporter 5 (KCC2) as an important modulator of neural circuits ^38^. KCC2 plays an important role in inhibitory neurotransmission at the synaptic cleft and subsequently balances the excitatory/inhibitory (E/I) ratio. Although pharmacological KCC2 agonists can improve behavioral recovery after SCI, this improvement diminishes upon cessation of daily drug administration ^38^. Gene therapy is an effective tool for continuous expression of neuro-modulatory factors, such as KCC2, and circumvents the continuous modulation required for modification of the spinal neural circuit. AAV-mediated KCC2 overexpression, under the influence of a synapsin promoter, is shown to improve functional recovery without the risk of adverse off-target effects associated with pharmacological strategies ^38^.

### Suppression of inhibitory molecules

Transcriptional suppression of intrinsic inhibitory molecules can circumvent the inability of adult neurons to regenerate across the injured spinal cord. Short-hairpin RNA (shRNA) constructs are capable of therapeutically silencing the expression of inhibitory factors. For instance, phosphatase and tensin homolog (PTEN) is a tumor suppressor, which converts phosphatidylinositol-3,4,5-triphosphate (ROP3) to phosphatidylinositol-4,5-bisphosphate (RIP2). PTEN blocks the growth and extension of adult neurons, as it is known to inhibit axonal protein synthesis through negative regulation of the mechanistic target of rapamycin (mTOR) ^39^. The downregulation of mTOR both in adulthood and after axonal injury limits the regenerative potential of damaged neurons ^39^. PTEN deletion via an AAV-mediated Cre–LoxP system enables axonal regeneration in the mouse corticospinal tract following injury ^39, 40^. Additionally, ** shRNA-mediated suppression of PTEN increases the regrowth of the corticospinal tract injury ^41^.

### Enzymatic degradation of the glial scar

The secretion of CSPGs by reactive astrocytes and other glial and non-neural cells in the glial scar is one of the major barriers to axonal outgrowth and regeneration ^16^. Enzymatic degradation of CSPGs, using the bacterial enzyme chondroitinase ABC (ChABC), has been proven in preclinical studies to improve regeneration and functional recovery after SCI ^42, 43^. However, ChABC has a low half-life and limited thermal stability, which requires its repetitive administration to the spinal cord ^44^. Gene therapy is one of the delivery options for ChABC, as it avoids the need for repetitive invasive enzymatic infusion while still promoting neuroplasticity and functional improvement. The application of lentiviral constructs allows temporal control of ChABC expression under inducible promoters (e.g. TetON promoters), suggesting that long-term expression of ChABC is critical for the recovery of fine motor movement after cervical SCI ^45^. This paves the way for a future clinical trial for ChABC gene therapy in SCI patients.

## Regenerative cellular therapies

**Figure 3.**
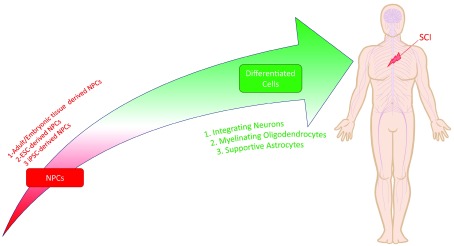
Neural progenitor cells as promising cells for the treatment of spinal cord injury. Neural progenitor cells (NPCs) are self-renewing, tripotent cells capable of differentiating into synaptically integrating neurons, myelinating oligodendrocytes, and supportive astrocytes. NPCs can be derived from adult or embryonic tissue sources or pluripotent cells like embryonic stem cells (ESCs) and induced pluripotent stem cells (iPSCs).

Cellular approaches hold promise as a regenerative therapy for SCI, as they address multiple facets of the injury pathophysiology concurrently ^46– 48^. Transplanted cells can replace lost neurons and glial cells, immunomodulate local and systemic environments, secrete critical neurotrophic factors, and produce a growth-permissive extracellular matrix to influence both cell survival and differentiation ^46, 47^. While numerous cell types, including mesenchymal stem cells, olfactory ensheathing cells, and Schwann cells, have been studied ^49^, the stem/progenitor cells with the potential to differentiate into neural cell lineages (neurons, astrocytes, and oligodendrocytes) are uniquely poised to regenerate the injured spinal cord. As a general term, these cells are referred to as neural stem/progenitor cells (NSPCs) or simply NPCs. NPCs are self-renewing, tripotent stem cells capable of differentiating into synaptically integrating neurons, myelinating oligodendrocytes, and astrocytes after transplantation into SCI ^44, 50– 53^ ( Figure 3). There are also studies that have used bipotent or unipotent cells that are lineage restricted ^54^. Oligodendrocytes and neurons differentiated from NPCs are capable of remyelinating denuded axons and can re-establish interrupted neuronal pathways via intra- and trans-segmental relay circuits ^50^. Our lab and others have shown that the two major mechanisms for functional recovery following NPC transplantation are 1) integration of NPC-derived neurons into disconnected circuits to relay neural signals and 2) myelination of denuded axons by NPC-derived oligodendrocytes ^53, 55– 57^.

NPCs have historically been derived from adult tissue sources (e.g. subventricular zone of the forebrain, subgranular zone of the dentate gyrus, and central canal in the spinal cord) or by differentiation of embryonic stem cells (ESCs) ^47, 48^. ESC-derived NPCs present ethical challenges, and the clinical derivation of NPCs from adult tissues for autologous transplantation is not feasible. However, exciting advances have facilitated safe NPC derivation from translationally relevant human induced pluripotent stem cells (hiPSCs). This is particularly advantageous, as iPSCs can be made from easily accessible autologous somatic cells (e.g. skin or blood) using non-viral techniques, which provides a clinically attractive approach to cell therapy ^47, 48^.

### Remyelination of the denuded axons

While recent work suggests the degree of endogenous remyelination by oligodendrocyte progenitor cells is higher than previously reported ^50^, it remains widely accepted that the functional benefits from these populations are limited owing to high rates of apoptosis and poor proliferation ^50^. Fortunately, it has been shown that transplanted NSPCs can differentiate to oligodendrocytes in the injured spinal cord to remyelinate denuded axons ^58, 59^ and concurrently promote preservation of endogenous myelin ^55, 60^. Importantly, we have found that this neurobehavioral effect is lost when adult NPCs are derived from myelin basic protein (MBP)-deficient *Shiverer* mice incapable of producing functional myelin ^61, 62^. Recognizing these preclinical discoveries, Lineage Cell Therapeutics is currently undertaking a phase I/II clinical trial for SCI employing human ESC-derived OPCs (clinicaltrials.gov identifier: NCT02302157). While this is an important first-in-human study, several limitations exist. First, the use of allogeneic ES-derived cells brings ethical, technical, and safety concerns. Human iPSC-derived cells would instead allow for the potential generation of autologous cell lines whilst avoiding ethical issues. Second, the bipotent OPCs cannot efficiently differentiate into neurons and, therefore, have limited potential to restore lost neuronal populations. In contrast, the proportion of mature oligodendrocytes that differentiate from typical tripotent NPCs is low in the injured spinal cord microenvironment, limiting functional recovery. To address this challenge, our lab has generated myelinating oligodendrogenic tripotent NPC (oNPCs) ^63^. oNPCs transplanted into rodents with SCI showed differentiation to both neurons and ensheathing oligodendrocytes with clear graft–host synapse formation and structural myelination ^53^. The transplanted cells also showed significant migration along the rostrocaudal axis and proportionally greater differentiation into oligodendrocytes. The oNPCs promoted perilesional tissue sparing and axonal remyelination, which resulted in motor function recovery. Our findings showed that biasing NPC differentiation along an oligodendroglial lineage represents a promising approach to promote tissue sparing, axonal remyelination, and neural repair post-SCI ^44, 53^.

### Restoring the interrupted neuronal pathways

NPC-derived neurons have the potential to integrate into endogenous neural networks and re-establish interrupted neuronal pathways. Despite recent progress, the level of graft–host integration and the degree of intra- and trans-segmental relay circuits regenerated by the transplanted neurons has been modest ^64– 66^. This is partly because of suboptimal differentiation of transplanted NPCs in the injured cord microenvironment to non-neuronal cells ^51, 55^ and partly because of the difference in the identity of transplanted NPCs within the spinal cord niche. Spinal cord trauma initiates a cascade of cellular and molecular changes that drastically alter the composition of factors and extracellular matrix proteins in the local niche ^67– 69^. These perturbations affect the fate determination of transplanted cells and impair their ability to effectively integrate with host tissue ^70^. In the post-injury niche, transplanted tripotent NPCs predominantly differentiate to astrocytes ^71, 72^. When endogenous adult NPCs proliferate in response to injury, the vast majority of the newly generated cells are glial fibrillary acidic protein-positive (GFAP+) astrocytes ^22, 73^. Similarly, when different types of NPCs are transplanted to a lesioned spinal cord microenvironment, they mainly differentiate into cells with an astrocytic phenotype ^71, 74^. Although differentiation to astrocytes may be important for regeneration ^15, 75^ to improve functional recovery after transplantation, it is important that NPC grafts can also differentiate into synaptically integrating neurons. Recently, we have shown that enhancing trophic support by overexpression of GDNF in the niche can greatly increase the differentiation profile of NPCs exposed to injured cord conditions—transitioning the profile from pro-astrocytic to pro-neuronal—which enhances functional recovery following SCI ^76^.

In addition to suboptimal differentiation, another reason for the poor integration of transplanted cells is a mismatch in cell identity. Native NPCs, along the entire rostrocaudal neural axis, possess a unique region-specific identity (e.g. forebrain, midbrain, cervical, thoracic, etc.) ^77^, which is accompanied by distinct neural differentiation in terms of channel composition, axonal projection pattern, and neurotransmitter phenotype. These distinct characteristics allow proper integration during development and in adulthood. Most of the NPCs currently used in preclinical and clinical studies possess a cortical brain identity, which is poorly suited for the spinal cord niche ^26, 78^. These cells terminally differentiate into neuronal cell subtypes (e.g. cortical, subcortical, or deep nuclear neurons), which do not reside in the spinal cord. Moreover, after SCI, there is a significant loss of spinal motor neurons and spinal interneurons (for example, propriospinal interneurons and Renshaw cells), which are likely required for a successful cell replacement approach. Evidence is now emerging that the limited integration of transplanted cells after SCI may be in part due to the graft’s discordant regional identity ^51^. Recent studies using hiPSC-derived cells have demonstrated that neural cell derivatives must possess a regional identity that mimics the respective endogenous CNS tissue in order to effectively engraft and regenerate. A plethora of research is currently focusing on differentiating stem cells into a V2a excitatory interneuron phenotype to optimize forelimb and hindlimb functional recovery following SCI ^79^. This appears to be a promising approach, as ESCs differentiated to express Chx10+ V2a interneurons have been shown to enhance functional recovery post-SCI ^80^. However, improved functional recovery using V2a differentiated stem cells does not equate to optimal functional recovery in all SCI contexts. The following are research questions that remain open in the field: what endogenous connections are these stem cell grafts forming, and which endogenous targets are best for restoring functional recovery to its original competency? To date, there are few to no publicly accessible data that elucidate which stem cells are interacting within an *in vivo* environment or which parameters are biasing their choice of neural connection (such as regional identity).

## Combinatorial strategies to enhance regeneration after spinal cord injury

The heterogeneity of SCI and the described pathophysiological complexity warrants the development of combinatorial and personalized treatment strategies ^81^. Over the years, multiple combinatorial approaches involving gene and cell therapies have been investigated for SCI. Here, we review the most recent findings employing rehabilitation training, neuromodulation, and biomaterials to enhance regenerative treatments and ultimately SCI recovery.

### Rehabilitation training

Rehabilitation training is the cornerstone of clinical interventions for SCI and has been demonstrated to improve the functional recovery of people with SCI ^82, 83^. Mechanisms behind the obtained recovery include the upregulation of neurotrophins (e.g. BDNF) ^84, 85^, activity-dependent neuroplastic changes of the spared networks ^86, 87^, increased regeneration, and axonal sprouting ^87, 88^. While rehabilitation offers functional benefits to individuals with SCI, improvements with this intervention alone are often limited. Despite the prevalence of rehabilitation training in clinical treatment protocols for SCI and its potentially synergistic mechanisms with other regenerative treatment strategies, studies of combinatorial treatments are still rare. Future preclinical studies should incorporate rehabilitation training paradigms to improve the SCI study model and simulate clinical conditions more closely ^89^.

Recently, a few studies have investigated combined rehabilitation and stem cell transplantation. One study demonstrated that following a T9 contusive injury, rats undergoing treadmill training (TT) and transplantation of NPCs showed superior functional recovery, graft survival, and remyelination when treatments were combined ^90^. In another study, following a T9 contusion injury in rats, transplantation of NPCs in combination with TT and ChABC enhanced functional recovery in the chronic phase of injury ^43^. Further investigations are needed to optimize such combinatorial treatment strategies.

### Biomaterial scaffolds

Biomaterial scaffolds have long been investigated as tools for the localized and prolonged delivery of drugs ^91^, bridging the injury site with a hospitable microenvironment for the regeneration of endogenous networks ^92, 93^, and the introduction of exogenous stem cells into a spinal microenvironment promoting cell survival, growth, and plasticity ^94^. A large range of biomaterials have been investigated for these goals such as the fibrin matrix ^95^, hyaluronan methylcellulose (HAMC) ^96^, and polyethylene glycol–gelatin methacrylate (PEG–GelMA) ^97^.

In applications related to stem cell therapies, design parameters such as the material, geometrical dimensions, shape ^98^, and mechanical properties of the scaffold ^99^ and the scaffold–stem cell interactions can influence the outcome ^100^. For instance, Leipzig and Shoichet investigated the effect of scaffold stiffness on the differentiation profile and proliferation of NPCs. They demonstrated that cultures in scaffolds with high stiffness were more oligodendrogenic compared with soft scaffolds that favored astrocytic and neuronal fates ^99^. Recent advances in the 3D printing of biomaterial scaffolds have made their precise morphological design possible. For instance, 3D-printed PEG–GelMA scaffolds can mimic the spinal cord morphology with microchannels located in its white matter region ^97^. Transplantation of such scaffolds seeded with NPCs into the spinal cord of rats with transection SCIs resulted in the columnar growth of exogenous NPC axons throughout the scaffold microchannels. Evidence of endogenous axonal growth into the scaffold was also reported ^97^.

Biomaterials are also promising tools for combining appropriate cell and drug treatment strategies. For instance, the combined transplantation of olfactory ensheathing cells and a bridge scaffold containing Schwann cells along with intrathecal administration of ChABC resulted in greater functional recovery compared to cell transplantation alone. These functional improvements were evident by both behavioral and neuroanatomical assessments ^100^. More recently, Nori and colleagues combined the transplantation of oligodendrogenic NPCs with biomaterial delivery of ChABC in a clip-contusion model of chronic thoracic SCI in rats ^44^. In this study, ChABC was delivered using a crosslinked methylcellulose (XMC) hydrogel capable of sustained ChABC release for 7 days ^101^. This combinatorial strategy led to superior functional recovery from SCI compared with stem cell transplantation alone. Fuhrmann and colleagues took this a step further and used biomaterials to deliver both stem cells and drugs into the spinal cord parenchyma. In this study, OPCs were delivered using injectable methylcellulose hydrogels conjugated with platelet-derived growth factor-A (PDGF-A) in a clip compression rat model of thoracic SCI. This strategy resulted in enhanced early survival of the grafted cells ^102^.

## Conclusions

The rate of SCI has been on the rise in the past few decades, and this condition currently constitutes the second leading cause of paralysis worldwide. Restoring sensorimotor and autonomic function for people with SCI can significantly improve their quality of life. Recent innovations in effective gene- and cell-based therapies have vastly improved the technical ability to induce regeneration in the spinal cord. However, successful clinical translation of these techniques requires further optimization. Combinatorial strategies can greatly enhance the implementation and functional outcomes of these regenerative treatments.

## Abbreviations

AAV, adeno-associated viral; ChABC, chondroitinase ABC; CNTF, Ciliary-derived neurotrophic factor; CSPG, chondroitin sulfate proteoglycans; EGF, Epidermal growth factor; ESC, embryonic stem cell; FGF2, Fibroblast growth factor 2; GDNF, glial-derived neurotrophic factor; HAMC, Hyaluronan methylcellulose; hiPSC, human induced pluripotent stem cell; IGF1, Insulin-like growth factor 1; KCC2, chloride potassium symporter 5; KLF, Krüppel-like factor; Omgp, Oligodendrocyte-myelin glycoprotein; MAG, Myelin-associated glycoprotein; MBP, Myelin basic protein; NG2, Neural/Glial antigen 2; Nogo A, Neurite outgrowth inhibitor A; NPC, neural progenitor cell; NSPC, neural stem/progenitor cell; oNPC, oligodendrogenic tripotent neural progenitor cell; OPC, oligodendrocyte progenitor cells; PEG–GelMA, polyethylene glycol–gelatin methacrylate; PTEN, phosphatase and tensin homolog; SCI, spinal cord injury; SDH, staggered double hemisection; shRNA, short-hairpin RNA; XMC: Crosslinked methylcellulose.
